# In-silico analysis of ribosome inactivating protein (RIP) of the *Cucurbitaceae* family

**DOI:** 10.1186/s13568-024-01718-z

**Published:** 2024-05-27

**Authors:** Quratulain Maqsood, Aleena Sumrin, Qurban Ali, Nazim Hussain, Saif Ul Malook, Daoud Ali

**Affiliations:** 1 Department of Centre for Applied Molecular Biology, University of the Punjab, Lahore, Pakistan; 2 Department of Plant Breeding and Genetics, University of the Punjab, Lahore, Pakistan; 3 Department of Entomology and Nematology, University of Florida, Gainesville, USA; 4 Department of Zoology, College of Science, King Saud University, PO Box 2455, Riyadh, 11451 Saudi Arabia

**Keywords:** Ribosome-inactivating proteins, Antifungal, Antibacterial, Antiviral

## Abstract

Ribosome-inactivating proteins (RIPs) are highly active N-glycosidases that depurinate both bacterial and eukaryotic rRNAs, halting protein synthesis during translation. Found in a diverse spectrum of plant species and tissues, RIPs possess antifungal, antibacterial, antiviral, and insecticidal properties linked to plant defense. In this study, we investigated the physiochemical properties of RIP peptides from the *Cucurbitaceae* family through bioinformatics approaches. Molecular weight, isoelectric point, aliphatic index, extinction coefficient, and secondary structures were analyzed, revealing their hydrophobic nature. The novelty of this work lies in the comprehensive examination of RIPs from the *Cucurbitaceae* family and their potential therapeutic applications. The study also elucidated the binding interactions of *Cucurbitaceae* RIPs with key biological targets, including Interleukin-6 (IL-6). Strong hydrogen bond interactions between RIPs and these targets suggest potential for innovative insilico drug design and therapeutic applications, particularly in cancer treatment. Comprehensive analysis of bond lengths using Ligpolt + software provides insights for optimizing molecular interactions, offering a valuable tool for drug design and structural biology studies.

**Supplementary Information:**

The online version contains supplementary material available at 10.1186/s13568-024-01718-z.

## Introduction

A ribosome-inactivating protein (RIP) is a protein that inhibits protein synthesis in the eukaryotic ribosome. This protein family refers to a large group of rRNA N-glycosylase-functioning proteins (Rolnik and Olas 2020; Wong et al. 2020). By transferring a particular adenine base from the sugar-phosphate backbone of 28 S rRNA, they inactivate 60 S ribosomal subunits. Plants and bacteria both have RIPs. Shiga toxins and ribosome inactivating proteins of type I (luffin and trichosanthin) and type II (agglutinin, ricin, and abrin) are members of the RIP family (Ghosh et al. 2021; Chanda et al. 2022). These toxins are structurally related to one another. RIPs have received to much attention due to their potential use as immunotoxins to treat cancer when combined with monoclonal antibodies (Citores et al. 2021a; Maqsood et al. 2021). Trichosanthin has also been shown to have potent anti-HIV-1 activity in macrophages and T cells (Jain et al. 2022). Determining the structure-function connections of RIPs has therefore become an important topic of study (Fapohunda et al. 2018). RIPs are now known to be structurally linked. The catalytic mechanism has been connected to a conserved glutamic acid that is positioned close to a conserved arginine that is also involved in catalysis (Soria et al. 2020; Mishra et al. 2022). Although only a tiny fraction of RIPs are harmful to humans when consumed, most plants used for human consumption, including rice, maize, and barley, include proteins from this family (Abbas et al. 2021; Citores et al. 2021a). They are said to protect plants against diseases and insects (de Vasconcelos et al. 2018; Lu et al. 2022). Numerous studies have been conducted to investigate the potential of RIPs as antiviral and anticancer medications due to their diverse biological features (Dhanraj et al. 2020; Cong et al. 2022; AHMAD et al. 2023; AMIN et al. 2023; Ullah et al.  2023a, b). The integration of RIPs in conjugates is one of the most capable applications of RIPs in medicine, notably in tumor therapy(Barkhordari et al. 2018; Chanda et al. 2022; Ullah et al. 2023a, b). Enzymatic RIPs in these conjugates attach to antibodies or tumor-targeting ligands, allowing them to infiltrate malignant cells. Because they are more effective and less expensive than commercially produced medications, new medication substitutes taken from plants should be discovered and developed to give pharmaceuticals with low adverse effects (Méndez-Cuesta et al. 2018; Kokorin et al. 2019; Bhatti et al. 2023). Six RIP peptide sequences from the *Cucurbitaceae* family were chosen for this study, and their main and secondary structures, physicochemical components, and functional features were evaluated using a number of bioinformatics methodologies (Matsumura and Urasaki 2020; Czajka et al. 2022; Fatima et al. 2023; Hassan et al. 2023). The findings might lead to a better understanding of the structure and method of action of RIP proteins and the precise development of innovative plant-based medicine substitutes (Ishtiaq et al. 2019; Demirel et al. 2022; Din et al. 2023).

## Methodology

The ribosome-inactivating protein (RIP) peptide sequences were downloaded using the National Center for Biotechnology Information’s (NCBI) (https://www.ncbi.nlm.nih.gov/) and UniProt databases (https://www.uniprot.com) (Kuznetsov and Bollin 2021). Six ribosome-inactivating protein (RIP) peptide sequences were chosen from the *Cucurbitaceae* family. Plants on the list include *Momordica charantia*, *Luffa acutangular*, and *T. kirilowii* trichosanthin, with accession numbers AAX20021.1, CCD28507.1, XP 022156707.1, CCD28521.1, ABY71834.1, and AAA34207.1. For further analysis, all peptide sequences were saved in FASTA format.

### Physiochemical properties

The ExPASy protparam tool (https://web.expasy.org/protparam/) was used to investigate the plant’s physiochemical features, such as its molecular weight, isoelectric point, positive and negative R groups, instability index, extinction coefficient, grand average hydropathy (GRAVY) and aliphatic index (Demirel et al. 2022). In addition to physiochemical properties, protein sequences for amino acid compositions were found (Table S1: Supplementary material tables).

### Secondary structure and functional analysis

Helical wheel plots were utilized to visualize and investigate the predicted transmembrane helices (Waterhouse et al. 2018). SOPMA was used to determine the secondary structural properties of the ribosome-inactivating protein (RIP) as shown in Table S2 (Supplementary material tables).

### Multiple sequence alignment and phylogenetic analysis

After the sequences of the chosen plants were aligned using UniProt’s Multiple Alignment tool (https://www.uniprot.org/align/), a phylogenetic tree was generated to determine the similarities and differences between these plants and is presented in the findings (Dougherty and Hudak 2022).

### Docking for insilico drug designing

Ribosome-inactivating protein dock with tumor receptor Interleukin-6 of these expressed in different tumor molecules. The docking were preformed by using PyRx multiple ligand docking software for drug design. Futher the validation of protein-ligand were done through ligPlot https://www.ebi.ac.uk/thornton-srv/software/LigPlus/.

## Results

**Fig. 1:**
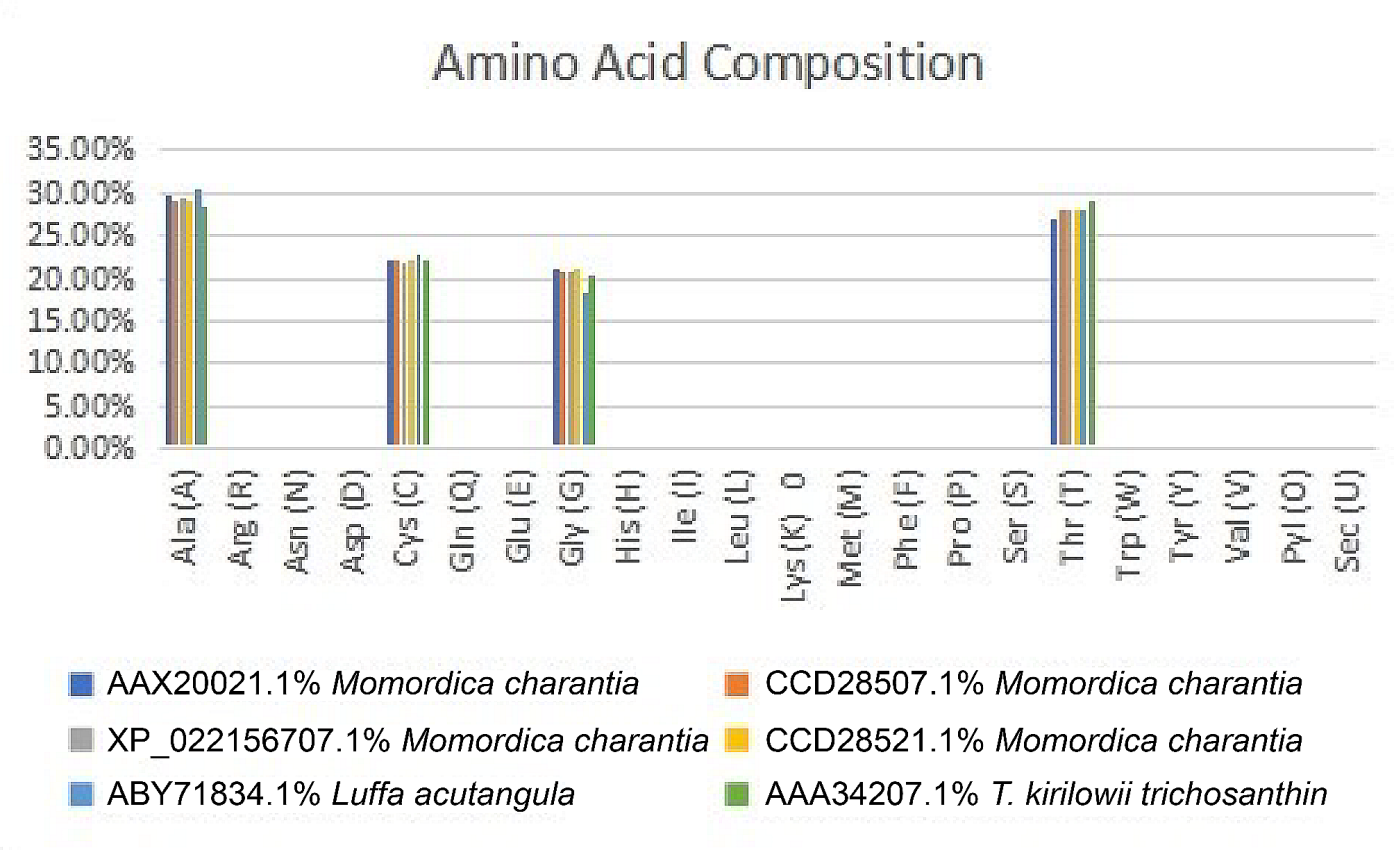
Amino acid composition of Ribosome inactivating protein of *Cucurbitaceae* family

**Fig. 2:**
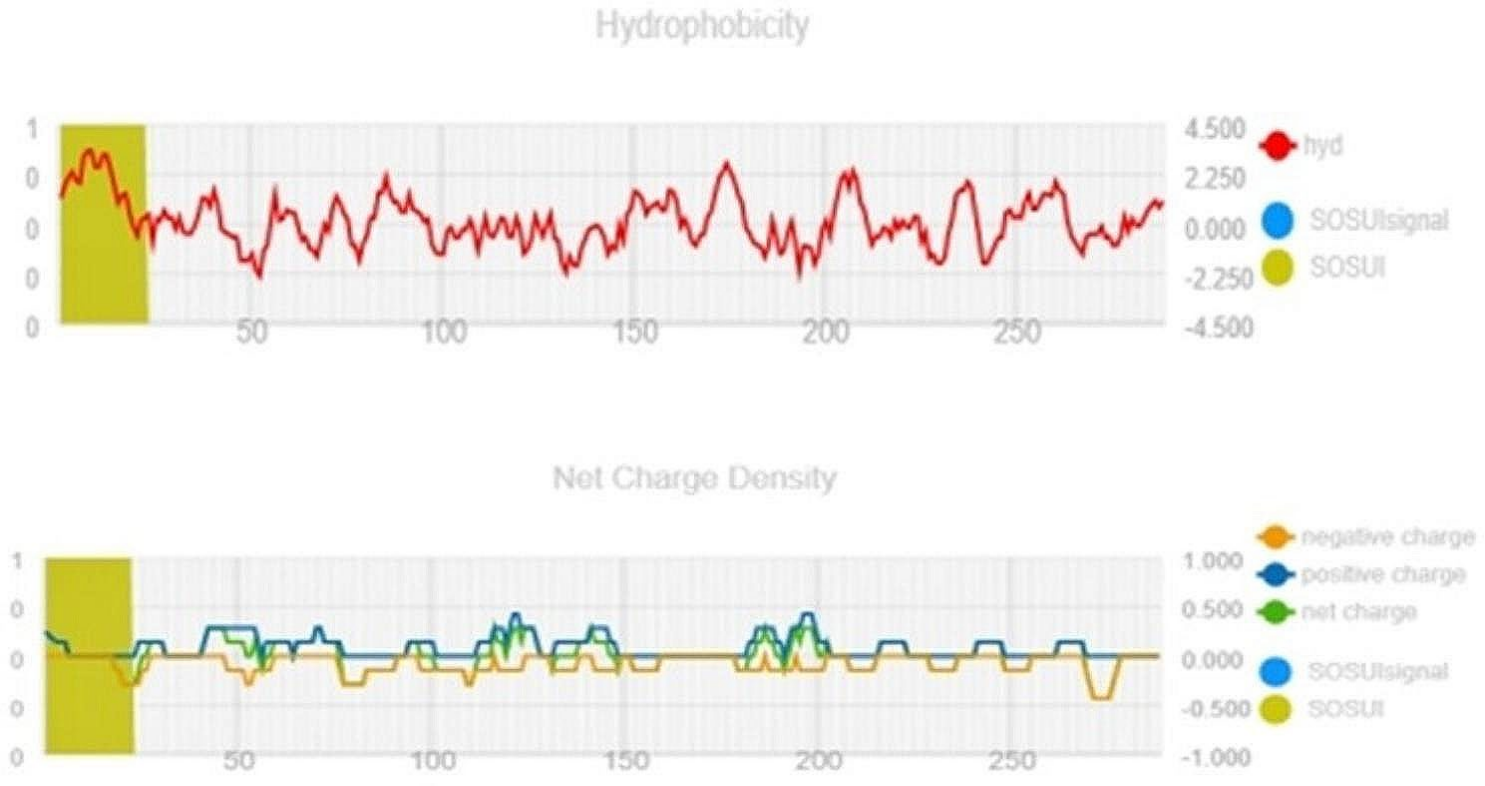
Graph showing hydrophobicity index and net charge density of RIP

**Fig. 3:**
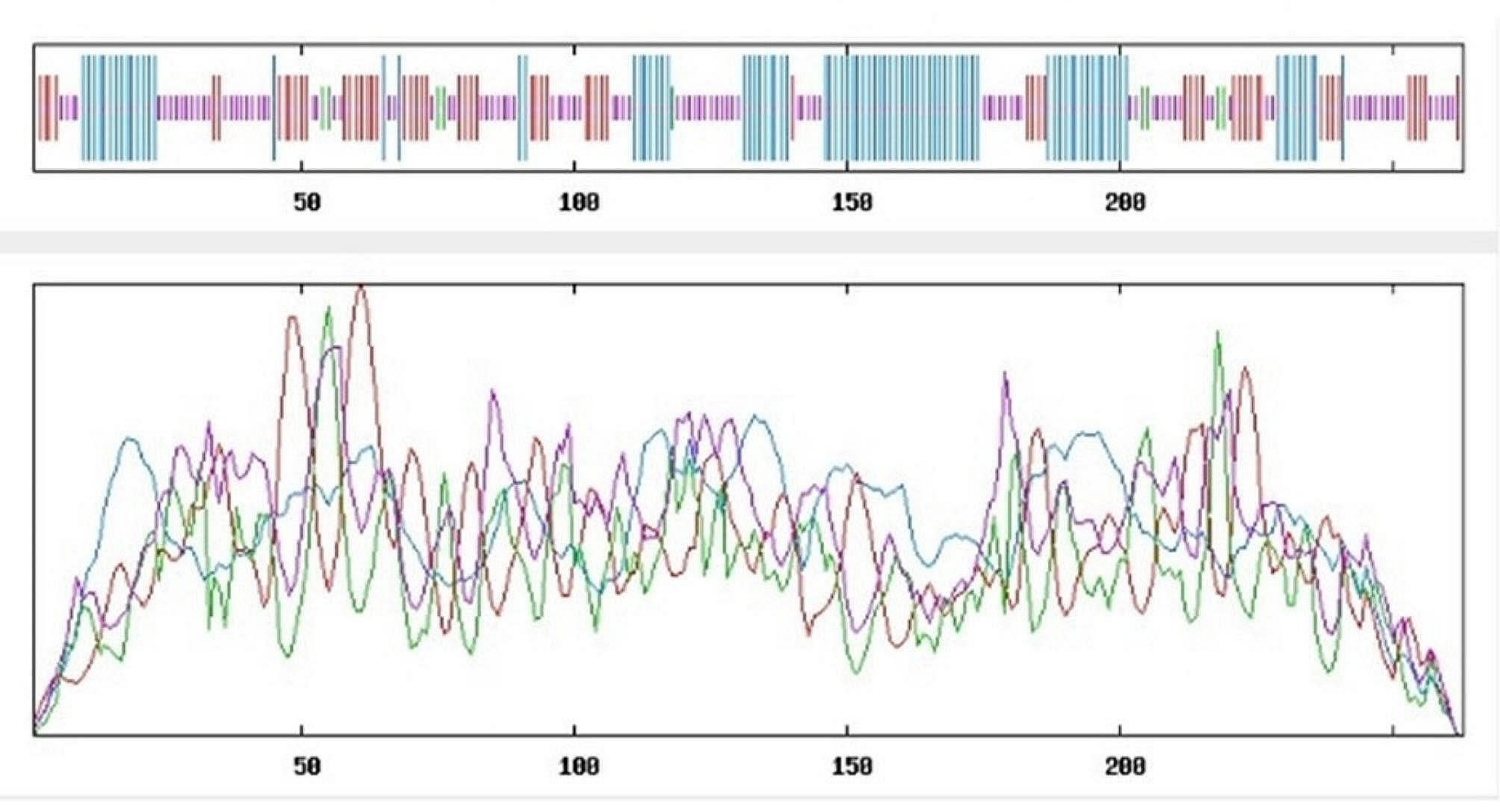
Secondary structure prediction of ribosome inactivating protein (RIP) of family *Cucurbitaceae * using standard parameters

The sequence of RIP, a *Cucurbitaceae* family anticancerous peptide retrieved from UniProt, was analyzed. To analyze the primary structure and compute a number of parameters, the ExPasy ProtParam tool was employed as shown in Table S1 (Supplementary material tables) (Rezaei-Moshaei et al. 2020; Kashyap et al. 2022). As indicated in Fig. 1 the protein is notably rich in Alanine (Ala), Cysteine (Cys), Glycine (Gly), and Threonine (Thr). This composition provides insights into the potential structural and functional characteristics of the protein within the context of its biological significance (Figs. 2 and 3).

**Fig. 4:**
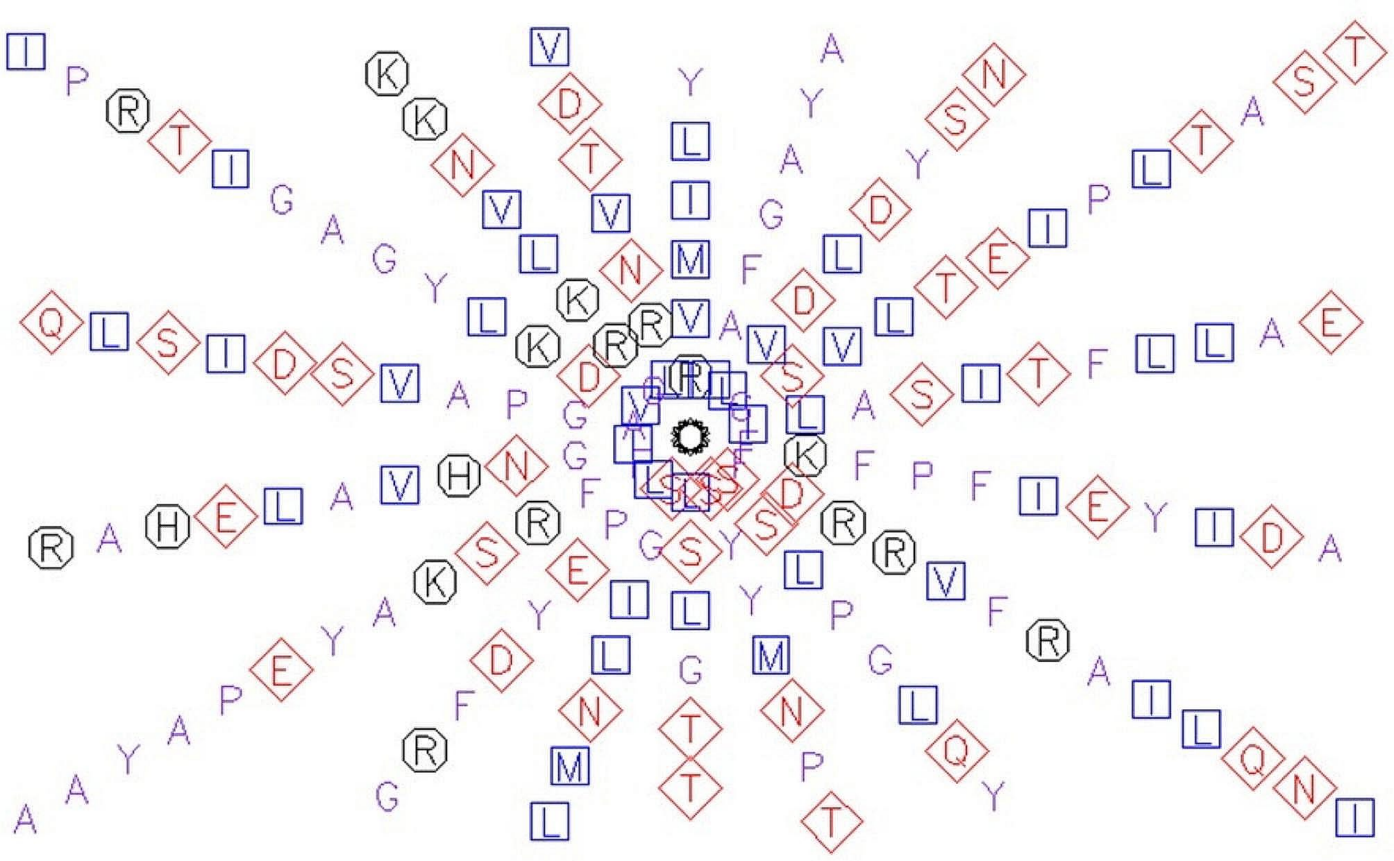
EMBOSS Pep wheel representation of predicted helix of ribosome inactivating protein (RIP)

Specifically, it focuses on the extinction coefficient (EC) values determined at 280 nm for the RIP peptides. Notably, all six protein sequences of RIP peptides exhibit a consistent EC range between 11,750 and 12,000 M^−1^ cm^−1^, with the exception of the ribosome inactivating protein (Fig. 4). The uniformity in EC values suggests a common characteristic among the RIP peptides, emphasizing their potential structural similarities or shared biochemical properties. The mention of the specific wavelength (280 nm) indicates the standard measurement conditions for assessing protein concentration and purity based on absorbance.

**Fig. 5:**
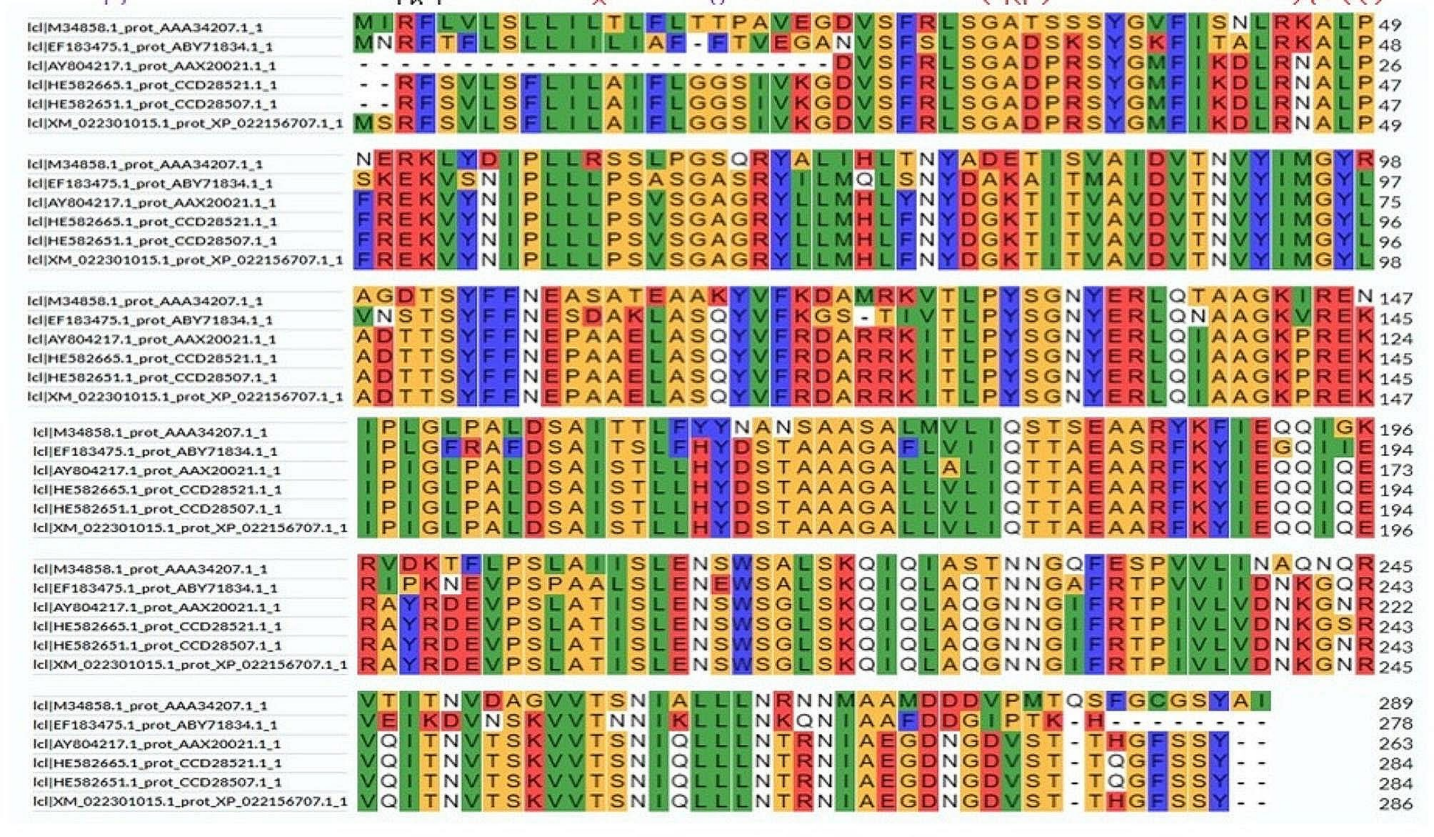
Alignment of amino acids in ribosome inactivating protein (RIP) protein in members of *Cucurbitaceae* family

The analysis reveals that approximately 37% of the secondary structure consists of a random coil, indicating regions lacking a defined regular structure. Additionally, around 34% of the protein adopts an alpha helix conformation, suggesting a substantial portion of organized helical structures within the RIP (Fig. 5). This information provides valuable insights into the protein’s overall structural composition, sheds light on its potential functional characteristics and contributes to a comprehensive understanding of RIP secondary structure features.

**Fig. 6:**
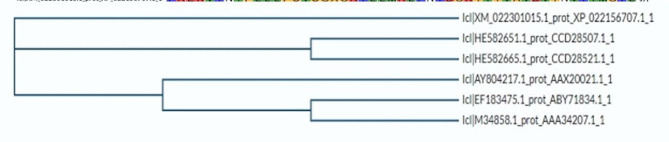
Phylogenetic tree of RIP protein in members of *Cucurbitaceae fa*mily

The alignment provides a visual representation of the similarities and differences in the amino acid sequences of RIP proteins within this plant family (Fig. 6). This information is crucial for understanding the evolutionary relationships and conserved regions among RIP proteins in *Cucurbitaceae*, offering insights into their functional and structural aspects.

The tree provides a visual depiction of the genetic relatedness and divergence among RIP proteins within this plant family. Clustering patterns and branch lengths in the tree offer insights into the evolutionary history and potential common ancestry of RIP proteins in *Cucurbitaceae*. This phylogenetic analysis aids in understanding the diversity and evolutionary dynamics of RIP proteins, contributing to a broader comprehension of their roles and adaptations within the *Cucurbitaceae* family (Fig. 6).

**Fig. 7:**
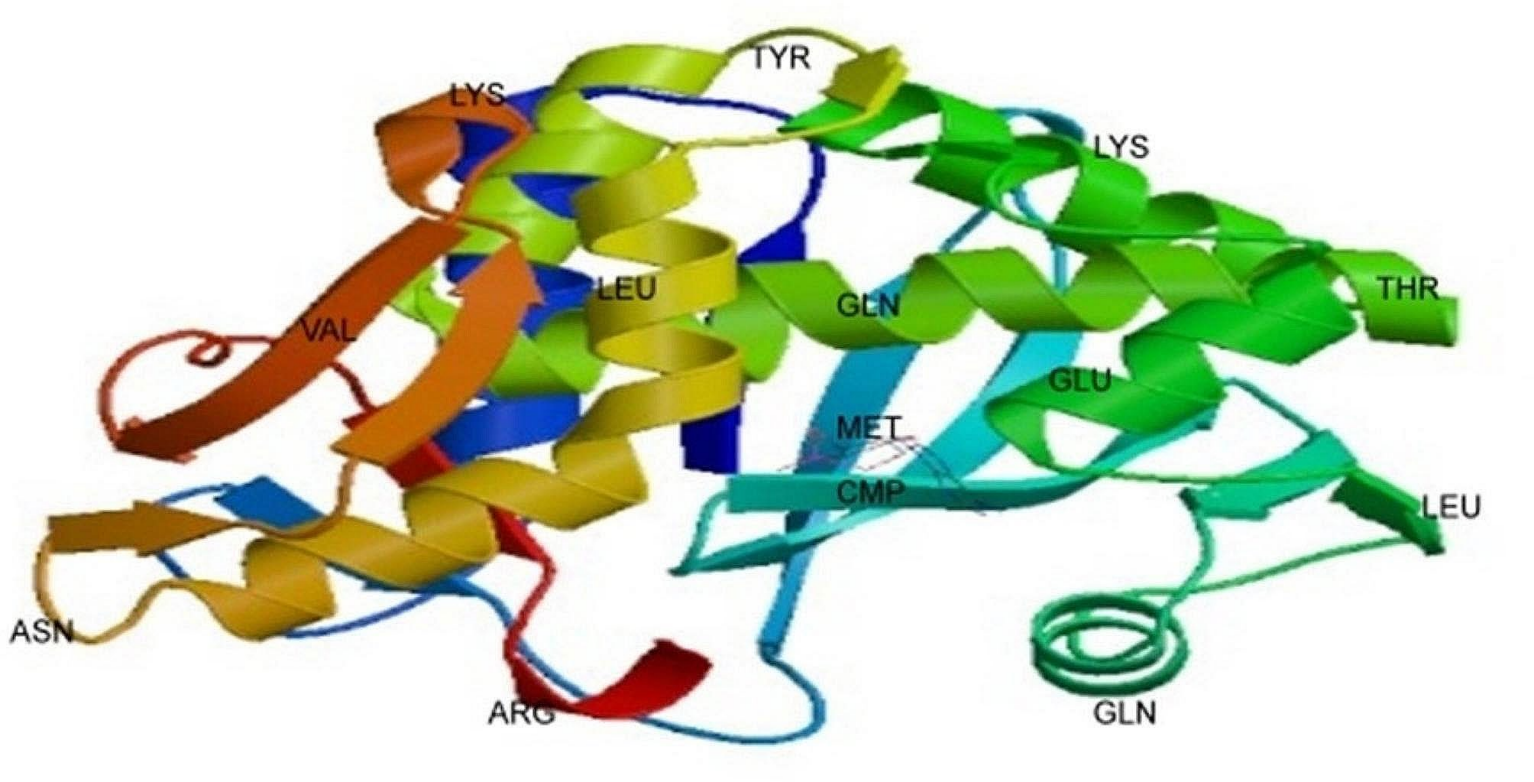
3D model of ribosome inactivating protein (RIP) of *Cucurbitaceae* family by using Swiss model

The model provides a three-dimensional representation of the protein’s structure, offering insights into its spatial arrangement and potential functional domains. This molecular visualization is crucial for understanding the structural characteristics of RIP in *Cucurbitaceae* (Fig. 7).

**Fig. 8:**
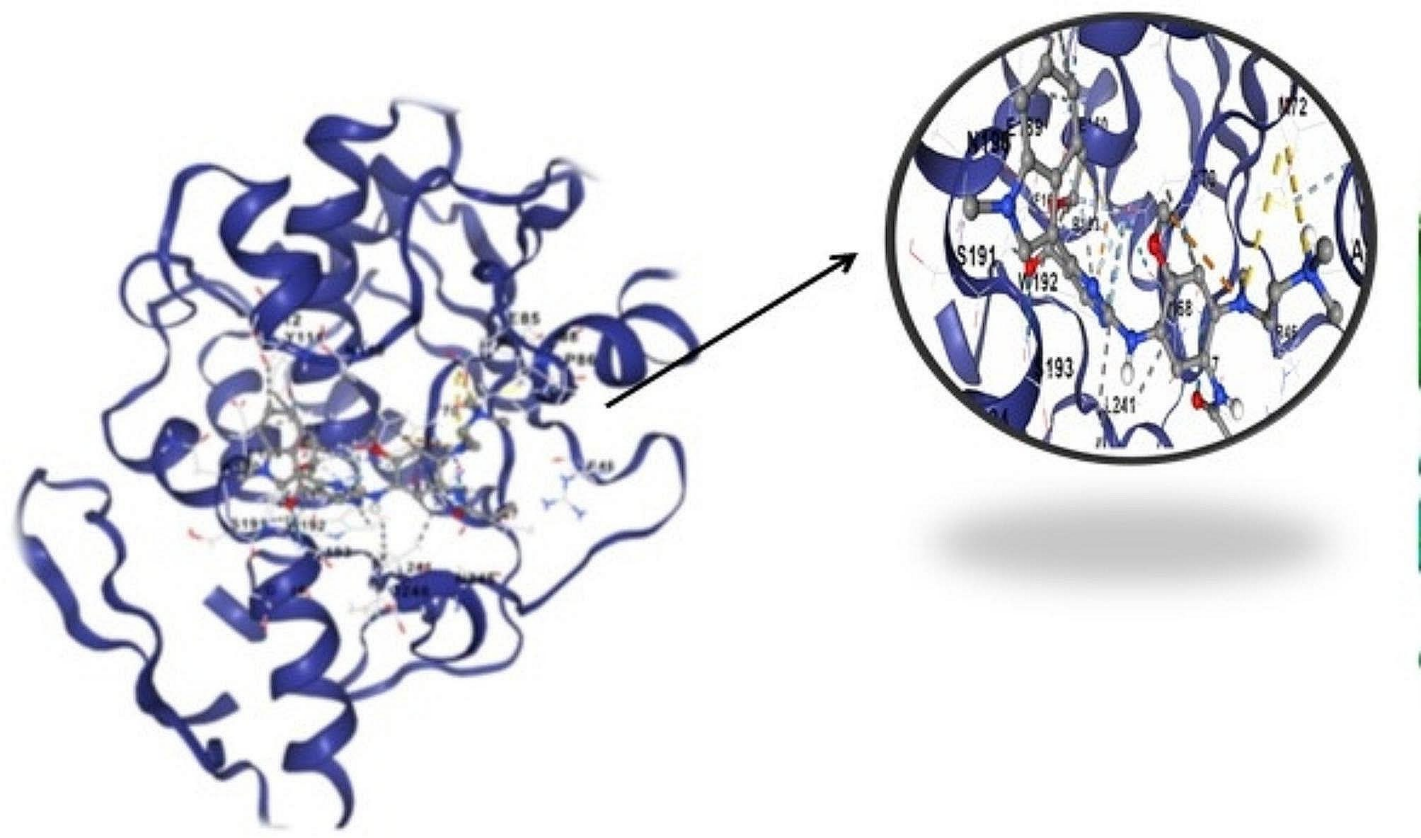
Docking of ribosome inactivating protein of *Cucurbitaceae* with Interleukin-6. RIPs show very strong Hydrogen-bond interaction with ligand IL-6

**Fig. 9:**
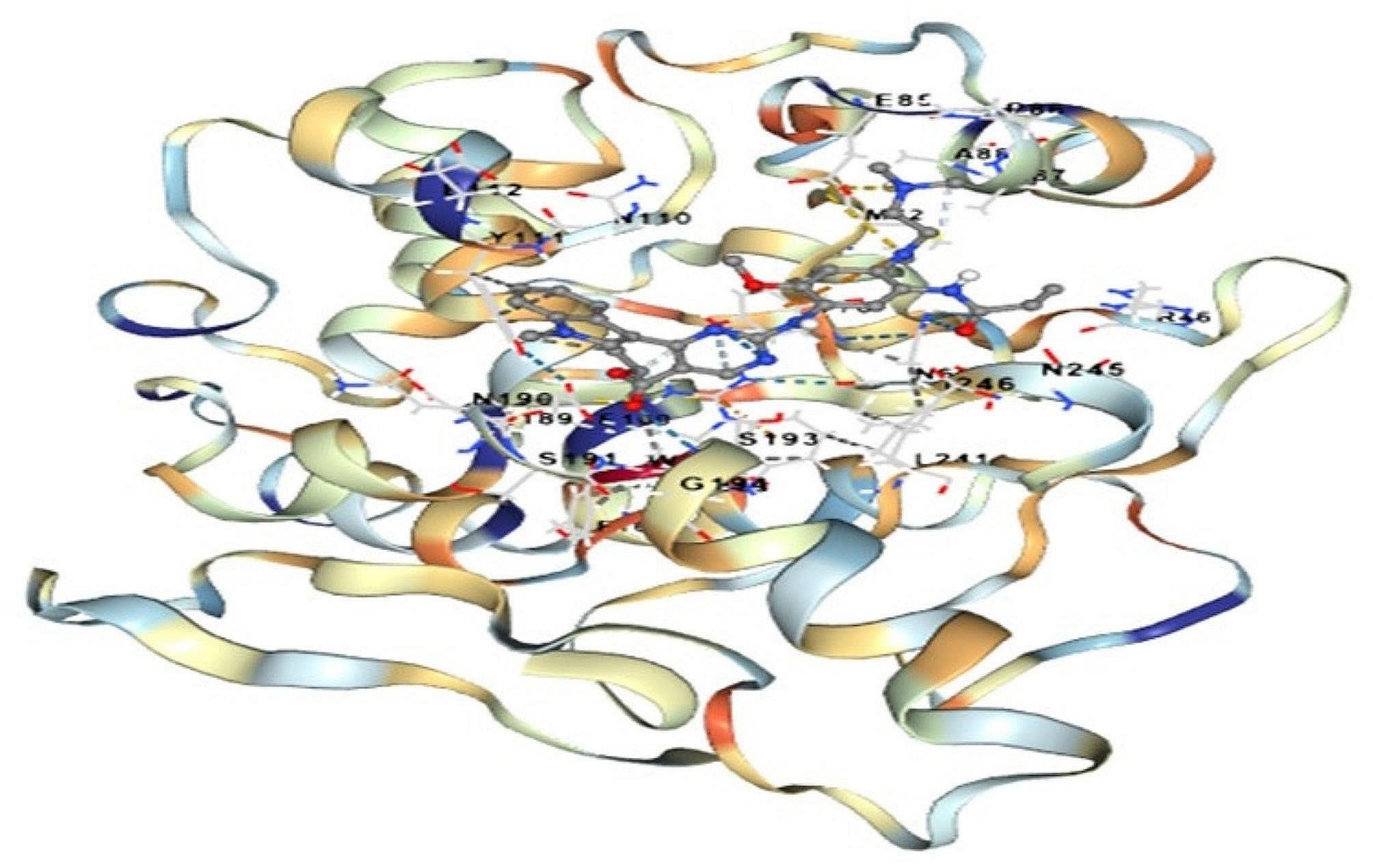
In the provided figure, a computational model illustrates the docking interaction between a ribosome-inactivating protein (RIP) derived from the *Cucurbitaceae * family and the epidermal growth factor receptor (EGFR). The model reveals a strong interaction characterized by multiple hydrogen bonds formed between the RIP and EGFR. This suggests a potential high-affinity binding between the two molecules, with significant implications for understanding their biological interactions, potentially for therapeutic or research purposes

The Fig. 8 illustrates a molecular interaction between RIPs and IL-6, emphasizing the presence of very strong hydrogen-bond interactions between the two molecules. This docking study provides valuable insights into the potential binding affinity and specific molecular interactions between RIPs and IL-6, shedding light on the possible roles of RIPs in modulating IL-6 activity or function. The information derived from this figure is essential for understanding the molecular mechanisms underlying the interaction between RIPs and IL-6, with potential implications for therapeutic or functional studies. The Fig. 9 displays green dots, indicating hydrogen bond interactions, which signify robust binding between the ligand and the protein. The visualization of these interactions provides a qualitative assessment of the strength and specificity of the binding between the two molecules. Hydrogen bond interactions are essential in molecular docking studies as they play a significant role in determining the strength and specificity of binding between a ligand and a protein. These interactions contribute to the stability of the ligand-protein complex, influencing the binding affinity and, consequently, the effectiveness of the ligand as a potential drug candidate. By visualizing hydrogen bond interactions in Fig. 9, we can qualitatively assess the robustness of the binding between the ligand and the protein, providing valuable insights for drug design and development.This analysis using PyMol contributes to the validation of the docking results, reinforcing the reliability of the predicted molecular interactions between the ligand and the protein in the complex.

**Fig. 10:**
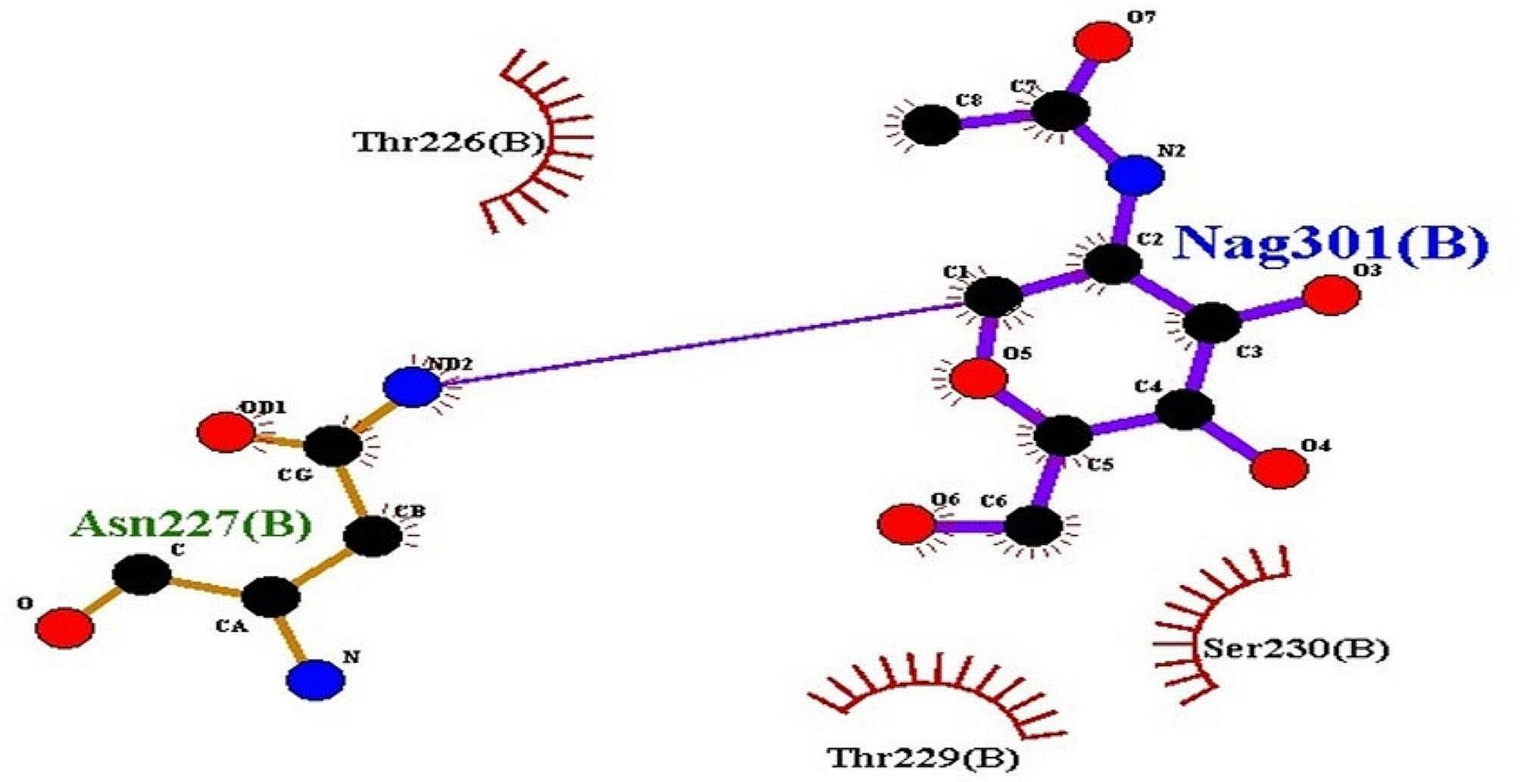
This figure depicts a visualization of bond lengths obtained using Ligpolt+ software. In the diagram, purple lines represent the bonds associated with the ligand molecule. Meanwhile, orange lines denote non-ligand interactions, particularly those involving protein residues to which the ligand forms hydrogen bonds. The transition from purple to red lines signifies the presence of hydrogen bonds, with the color gradient indicating the bond lengths. Notably, non-ligand bonds appear as thinner, orange lines, offering a clear distinction between different types of interactions within the molecular structure

In the diagram, purple lines represent the bonds associated with the ligand molecule. Meanwhile, orange lines denote non-ligand interactions, particularly those involving protein residues to which the ligand forms hydrogen bonds (Fig. 10). The transition from purple to red lines signifies the presence of hydrogen bonds, with the color gradient indicating the bond lengths. Notably, non-ligand bonds appear as thinner, orange lines, offering a clear distinction between different types of interactions within the molecular structure.

## Discussion

The presence of an ionic connection in the relevant RIP peptide is demonstrated by the presence of 29.90% ALa (A) residues in RIP, as shown in Fig. 1. It was chosen because the solubility is almost minimal at that estimated isolelectric point (pI) mobility under an electro-focusing system(Nikulin et al. 2018). To show this, the isoelectric point (pI), a measurement that indicates whether a molecule has positive or negative charges. The computed pI values of all peptides, which were frequently between pI < 7 and pI > 7, revealed that they were either acidic or had about equal negative and positive charges (Bidkar et al. 2014). Each ribosome inactivating protein RIP peptide’s estimated pI value reveals whether it is acidic or basic in nature, with pI < 7 suggesting that the peptides are acidic. The pI value for the whole six-protein sequence of the ribosomal -inactivating peptide, or RIP peptide, is 5.1, indicating that RIP peptides are acidic. The pI value of a particular demanding protein was determined to be useful in the construction of buffer systems throughout the purification process employing isoelectric focusing methods (Arif et al. 2022). The EC values are frequently useful for the quantitative investigation of protein-ligand and protein-protein interactions in any solution. Without the extinction coefficient, UV spectral methods cannot be used to analyze proteins (Ghosh et al. 2021). ExPasy protparam calculated the instability index (II) value of the ribosome inactivating protein (RIP) peptide, which provides an indication of the protein’s in vitro stability as shown in Table S1. If a protein’s instability index is less than 40, it is deemed stable. The ribosome inactivating protein (RIP) peptide’s six protein sequences have an instability index ranging from 52.58 to 48.89 (Kokorin et al. 2019). The aliphatic index, or AI, is the proportional volume of a protein occupied by aliphatic side chains (A, V, I, and L), and it is thought to improve the thermal stability of globular proteins (Dougherty and Hudak 2022). The protein from Luffa acutangula has a high aliphatic index of 30.59 and is found in all six protein sequences of the ribosome inactivating protein (RIP) peptide, which has an aliphatic index ranging from 28.39 to 30.59. The Grand Average Hydropathy (GRAVY) value of a protein is calculated by summing the hydropathy values of all its amino acids as shown in Table S1, then dividing the result by the number of residues in the protein sequence (Li et al. 2020; Ragucci et al. 2021). The GRAVY indices of the ribosome inactivating protein (RIP) proteins range from 0.78 to 0.854, indicating that virtually all of the RIP proteins studied in this study are hydrophobic (Russo et al. 2015; Moghadam et al. 2016). However, RIP protein functional and physicochemical characterization was also carried out, as well as transmembrane or TM region identification, followed by the prediction of disulfide bonding pairs, and so on. Except for the T. kirilowii trichosanthin RIP peptides, all ribosome inactivating protein (RIP) sequences have two transmembrane helices (Wong et al. 2020; Pathanraj et al. 2021). Sequence alignment reveals that the signal peptide’s amino acids vary more than those of the mature peptide, as shown in Fig. 3. The sequence exhibits variation at 117 distinct locations. The secondary structures of ribosome-inactivating protein (RIP) were predicted using SOPMA (self-optimized prediction approach with alignment), which reliably predicts 100% of amino acids (Table S2 for a detailed description of the secondary structure prediction) (Rotskaya et al. 2021). The amino acids in all six protein sequences are found in alpha helices, beta turns, extended strands, and random coils, but the *Cucurbitaceae* family’s RIP amino acids are primarily composed of random coils and alpha helices, accounting for 33.46% and 39.10% of all selected protein sequences, respectively. Random coils predominated among the estimated secondary structure features, followed by alpha helix, extended strands, and beta turns for all sequences, whereas all other secondary structure features, such as the 310 helix, pi helix, ambiguous states, bend area, and beta bridge, were not observed (Pizzo et al. 2018). Random coli is the most common secondary structural component in all RIP peptides (Cc). Secondary structures were predicted using default parameters (Windows width: 17, similarity threshold: 8, and number of states: 4) (Nivetha et al. 2021). The helix of the ribosome-inactivating protein, is viewed with the EMBOSS pepwheel, as shown in Fig. 4. Hydrophobic residues include V, G, A, L, S, and F in black squares, as well as I in violet letters. Analyzing three-dimensional protein structures makes it straightforward to learn about protein properties, functional features, and sequencing. When a precise, experimental three-dimensional protein structure is unavailable for comparison, protein homology models play a larger role in biological research analysis and experiment design. However, it is possible to predict the 3D protein structure from amino acid sequences by utilizing information from numerous web-based protein homology modeling online services with all conceivable levels of complexity in protein structures. Because of changes in protein structures brought about by evolution, the three 3D structures exhibit similarities and contrasts. The 3D protein structure was simulated using the Swiss model for ribosome-inactivating protein (RIP) as shown in Fig. 5 (Pathanraj et al. 2021). The phylogeny of these plants shows that there is a close resemblance between the same group of ribosomes-inactivating protein of the family *Cucurbitaceae*. As shown in thephylogenetic tree *Luffa acutangula* (ABY71834.1%) and *T.kirilowii trichosanthin* (AAA34207.1%) are more close related to each other (Dougherty and Hudak 2022; Fatima et al. 2023).

In this article, we have delved into the intriguing world of ribosome-inactivating proteins (RIPs) and their versatile roles in molecular interactions and drug design. As shown in Fig. 8 the results of docking interactions between a RIP from the *Cucurbitaceae* family and Interleukin-6 (IL-6). What stood out in this interaction was the remarkable strength of the hydrogen bonds formed between RIPs and IL-6. This observation is of paramount significance as RIPs have previously exhibited potent anticancer effects against a variety of cancer cell types. The strong binding affinity indicated by the hydrogen bonds implies that RIPs could play a pivotal role in insilico drug design targeting IL-6, potentially opening up novel avenues in cancer treatment. As shown by the results the bond-interaction between a *Cucurbitaceae* -derived RIP and IL-6. The computational model revealed a robust interaction characterized by multiple hydrogen bonds. This has significant implications in the realm of therapeutic applications and scientific research, especially concerning IL-6 associated diseases, such as cancer. The strong binding observed between RIPs and IL-6 suggests the potential for therapeutic interventions aimed at modulating IL-6-related pathways. In Fig. 9, the computational model showcases the docking interaction between a ribosome-inactivating protein (RIP) from the *Cucurbitaceae* family and the epidermal growth factor receptor (EGFR), revealing multiple hydrogen bonds that suggest a high-affinity binding. These findings not only shed light on the molecular interactions between RIPs and EGFR but also hint at their potential therapeutic or research applications, highlighting the significance of studying such interactions in drug discovery and development. The visualization of bond lengths using Ligpolt + software. This comprehensive depiction of molecular interactions is invaluable in understanding the strength and specificity of these bonds as shown in Fig. 10. The color-coded representation, distinguishing ligand bonds, non-ligand interactions, and hydrogen bonds, facilitates drug design and structural biology studies. Such in-depth analysis of bond lengths enhances our ability to design molecules with optimal binding properties. In conclusion, promising potential of ribosome-inactivating proteins in molecular interactions and drug design. These findings have implications that extend beyond the laboratory, holding the promise of innovative cancer therapies and providing essential insights for structural biology studies. The robust binding affinities observed in the context of RIP interactions with IL-6 underscore the significance of these proteins in targeting cancer-related pathways. Furthermore, the visualization of bond lengths using Ligpolt + software equips researchers with a powerful tool to fine-tune drug design efforts. This work paves the way for further research into the applications of RIPs and their role in the development of novel therapeutic agents.

### Supplementary Information

Supplementary material 1

Supplementary material 2

## Data Availability

All data generated or analyzed during the study are included in the manuscript.
