# Supplementary material for: In-silico analysis of ribosome inactivating protein (RIP) of the Cucurbitaceae family
Source: AMB Express. 2024 May 27;14:61. doi: 10.1186/s13568-024-01718-z (PMC11130107; doi:10.1186/s13568-024-01718-z)
Supplement: Supplementary file 1 — Supplementary material 1 [file 13568_2024_1718_MOESM1_ESM.docx]

**Supplementary materials Tables**

**Table S1: Provides information on the physicochemical properties of Ribosome-inactivating protein (RIP) peptides analyzed using Expasy's ProtParam tool**

| Properties | AAX20021.1 % | CCD28507.1 % | XP_022156707.1 % | CCD28521.1 % | ABY71834.1 % | AAA34207.1 % |
| --- | --- | --- | --- | --- | --- | --- |
|  | *Momordica charantia* | *Momordica charantia* | *Momordica charantia* | *Momordica charantia* | *Luffa acutangula* | *T.kirilowii trichosanthin* |
| Amino acid | 789 | 855 | 861 | 855 | 837 | 870 |
| Molecular Weight (Da) | 65746.8 | 71529.2 | 72013.7 | 71515.2 | 70562.5 | 73202.1 |
| isoelectric point | 5.11 | 5.1 | 5.1 | 5.1 | 5.1 | 5.1 |
| Negative residue | 0 | 0 | 0 | 0 | 0 | 0 |
| Positive residue | 0 | 0 | 0 | 0 | 0 | 0 |
| Extinction Coefficient | 10875 | 11750 | 11750 | 11750 | 12000 | 12000 |
| Instability Index | 52.58 | 52.09 | 51.63 | 51.99 | 55.05 | 48.89 |
| Aliphatic Index | 29.91 | 29.12 | 29.38 | 29.01 | 30.59 | 28.39 |
| GRAVY | 0.821 | 0.798 | 0.795 | 0.795 | 0.854 | 0.78 |

**Table S2:** **Calculated secondary structure features of Ribosome-inactivating protein (RIP) using the SOPMA method**

| Accession No. | Features of Secondary structure | | | |
| --- | --- | --- | --- | --- |
|  | Alpha helix % | Beta turn % | Extended Strand % | Random coil % |
| AAX20021.1 | 33.46% | 3.42% | 23.95% | 39.16% |
| CCD28507.1 | 33.45% | 3.17% | 25.70% | 37.68% |
| XP_022156707.1 | 32.87% | 3.15% | 25.17% | 38.81% |
| CCD28521.1 | 33.10% | 3.87% | 24.65% | 38.38% |
| ABY71834.1 | 34.17% | 6.47% | 27.34% | 32.01% |
| AAA34207.1 | 36.33% | 5.19% | 19.72% | 38.75% |
